# Editorial: Childhood obesity: prevention, management and new insight in pathophysiology

**DOI:** 10.3389/fendo.2023.1200978

**Published:** 2023-04-18

**Authors:** Domenico Corica, Iwona Ben-Skowronek, Rade Vukovic, Malgorzata Wasniewska

**Affiliations:** ^1^ Department of Human Pathology in Adulthood and Childhood, University of Messina, Messina, Italy; ^2^ Department of Pediatric Endocrinology and Diabetology, Medical University in Lublin, Lublin, Poland; ^3^ Department of Paediatric Endocrinology, Mother and Child Health Care Institute of Serbia “Dr Vukan Cupic”, Belgrade, Serbia; ^4^ School of Medicine, University of Belgrade, Belgrade, Serbia

**Keywords:** obesity, children, epidemiology, pathogenesis, adipokine, obesity-related complication

Childhood obesity (CO) has become a major health problem worldwide and its incidence is steadily increasing (1, 2). Obesity is associated with a wide spectrum of both cardio-metabolic and psychological-behavioural complications that can occur as early as childhood and adolescence and can lead to a deterioration in the quality and perspective of adult life (3).

This Research Topic includes thirteen studies evaluating different aspects of CO. Specifically, six studies, both experimental and review studies, evaluate epidemiological aspects and risk factors related to childhood obesity; four studies analyze possible biochemical markers related to the pathogenesis of obesity-related complications; three studies evaluate psychological-behavioural aspects in children and adolescents with CO.

In recent decades, lifestyle changes, mainly characterized by an increase in sedentariness and unhealthy eating habits, are among the main culprits for the growing incidence of CO. In this context, the COVID-19 pandemic has undoubtedly amplified these aspects worldwide, further accelerating the spread of CO. Yang et al. evaluated the real-world national trends of obesity prevalence of Chinese children between the years 2017-2021 to assess the impact of COVID-19 pandemic on pediatric obesity. Through the collection of hospital and parent-reported data, the authors assessed the prevalence of obesity/overweight in the pre-pandemic and pandemic periods and changes in the BMI z-score during the COVID-19 lockdown in a large number of subjects. Authors demonstrated a relatively stable trend in the prevalence of CO in the pre-pandemic period and a significant increase in the prevalence during the COVID-19 pandemic; in particular, a high increase in the BMI z-score among primary and secondary school children, especially in specific regions, was documented. The authors concluded that these data support a targeted intervention, including through mobile growth assessment based on parent-reported, to prevent the spread of obesity especially during pandemic periods in China.

The spread of CO over the years has been accompanied by a lowering of its onset age. The diagnosis of simple obesity is often made as early as the first 3-5 years of life. However, in this context, attention should always be kept on those possible cases of obesity secondary to other causes, such as genetic obesities. This is the goal of the multicenter research project by Mierzwa et al. who aimed to create a Polish database of severely obese children and adolescents and to assess the prevalence of monogenic forms of obesity in this cohort, with a focus on abnormalities in the leptin-propylanocortin pathway. Another aspect related to causes of secondary obesity is assessed by the research of Hetman et al., who evaluate the application of standard or specific growth charts for early identification of growth disorders, including obesity, in children with Down syndrome.

CO, especially severe and early-onset obesity, is known to be related to an increased risk of developing cardio-metabolic complications, such as altered glucose and lipid profile, non-alcoholic fatty liver disease (NAFLD), hypertension. Abdominal obesity is one of the factors primarily related to these complications. Liu et al. assessed the change over time in the prevalence of abdominal obesity in a U.S. population, documenting that, although there was no significant increase in the prevalence of abdominal obesity in the entire population, some ethnic groups did show a significant increase, which therefore warrant further attention.

Due to the increasing incidence of CO, NAFLD has become the most common hepatopathy in pediatric age. The diagnostic gold-standard is liver biopsy, which, however, is burdened by several limitations, including invasiveness and costs. Therefore, ultrasonography, sometimes combined with elastography, is the most widely used noninvasive method to identify hepatic steatosis, although this method also has limitations (4). Marcinkiewicz et al. in their study, using liver ultrasound, identified NAFLD in approximately 23% of subjects in a pediatric population, with a higher prevalence among subjects with glucose intolerance. Therefore, liver ultrasonography can play a role, at least a preliminary one, in diagnosis and follow-up of NALFD in CO.

In addition to the diagnosis and subsequent tailored therapeutic approach to CO, the task of the scientific community and health professionals is to identify the causes promoting the onset of CO in order to implement preventive actions to curb this growing health problem. Factors influencing the onset of CO include both environmental factors (including nutritional aspects) and, in general, the individual’s lifestyle. However, the possible influence of pre-natal factors should also be considered. Several evidence showed that the origins of CO can be as early as maternal pregnancy. In a systematic review and meta-analysis, Yan et al. suggested that gestational hypertension and preeclampsia might be associated with obesity in the offspring. However, both possible prenatal (e.g., maternal factors) and postnatal confounding factors able to influence the onset of obesity and aspects related to hormonal adaptations of the fetus exposed to stressors, should always be considered.

A significant part of the scientific research concerning CO, is directed toward the investigation of new markers as predictors of obesity and its complications.

Cystatin C, a non-glycosylated protein filtered by the glomerulus, has been linked to obesity-related complications (e.g., NAFLD, vascular alterations) mainly in adult populations. In a Chinese cross-sectional study, Huo et al. analyzed the association between cystatin C and overweight or obesity in adolescence. In this study, authors suggested a potential role of serum cystatin C levels as an indicator of early obesity risk in adolescents, although further studies will be needed to confirm the preliminary results of this study.

To assess the risk of cardiovascular disease in obese children, Tumor Necrosis Factor Weak Inducer of Apoptosis (TWEAK), also known as Tumor Necrosis Factor (TNF) ligand superfamily member 12, probably involved in insulin resistance, may be used. Cluster of Differentiation 163 (CD163) is a macrophage-specific protein that has been identified as a scavenger receptor of TWEAK, promoting its degradation. CD163 is described as a strong predictor of type 2 diabetes in adults. In cardiovascular diseases, a high CD163/TWEAK ratio was found. The study by Escartin et al. is the first one to observe the evolution of these parameters in prepubertal children, supporting the hypotheses that these cytokines probably play a role in childhood obesity.

Endoplasmic reticulum (ER) stress proteins could play a role as early markers of metabolic alterations in childhood and adolescence. The results of a multicenter study from Italy (Antoniotti et al.) suggested the existence of an important link between ER stress and metabolic changes behind obesity complications even in pediatric age. These preliminary data suggest that Calreticulin (CALR) and PDIA3, two key molecules of ER stress, could be related to insulin resistance and altered lipid profile in pediatric obesity. These authors suggested that CALR and PDIA3 could be early markers of insulin resistance and dyslipidemia ER stress-related, useful to stratify patients at higher risk of further complications.

He et al. evaluated sex differences in insulin resistance-induced changes in metabolic and inflammatory markers in school-age children with overweight and obesity. These authors demonstrated an elevated white blood cell count and absolute neutrophil count in children with overweight and obesity, especially in girls, and a strong association of these parameters with HOMA-IR. Therefore, they suggested that these findings can be markers of insulin resistance.

Psychological-behavioral complications related to CO were also evaluated in this Research topic. Wojcik et al., in the original research project studied the prevalence of depressive symptoms and anxiety in adolescents with obesity and their caregivers. The results of the study showed that in childhood obesity, anxiety disorders are much more important than depressive disorders, both for patients and their parents. These authors concluded that childhood obesity, like any chronic disease, could have a significant impact on the emotional state of children and adolescents as well as the possibility of realizing interests and spending free time.

Second paper of this group is “A Brief Literature Review of the Role of Social Media in Body Image Shaping and Eating Patterns among Children and Adolescents” by Modrzejewska et al. The careful search of the literature had revealed only 8 articles for the analysis of this topic. The data analysis showed that social media may have a strong influence on the development of eating patterns and body image in children and adolescents, which in turn could be one of the risk factors for the development of obesity. Due to their great influence on youth, social media should be used as a resource for the prevention and treatment of obesity.

Finally, in a mini review Shi et al. evaluated mechanisms underlying the link between central precocious puberty and obesity, both from metabolic and cognitive-behavioral perspectives.

The studies in this Research Topic provide valuable evidence on several aspects regarding CO, providing input for further research on the topic. In conclusion, numerous factors (genetic, environmental, sociodemographic, behavioral, and perinatal) may contribute to the onset of obesity and its complications, although to date their exact role, interaction, and mechanisms involved in this process are not completely understood. Early intervention is an important strategy to prevent the onset of obesity, because there are crucial times during children’s growth and development when preventive efforts or therapeutic interventions can be most effective.

## Author contributions

All authors planned the outline of the editorial. DC, IB-S and MW drafted the manuscript. All authors contributed to manuscript revisions and approved the final version.
